# Efficiency of breeding olives for resistance to Verticillium wilt

**DOI:** 10.3389/fpls.2023.1149570

**Published:** 2023-02-22

**Authors:** Pedro Valverde, Diego Barranco, Francisco Javier López-Escudero, Concepcion Munoz Díez, Carlos Trapero

**Affiliations:** Department of Agronomy (Excellence Unit ‘María de Maeztu’ 2020-23), ETSIAM, University of Cordoba, Cordoba, Spain

**Keywords:** agronomical traits, breeding program, genotypes, new cultivars, *Olea europaea*, *Verticillium dahliae* (Kleb)

## Abstract

Olive trees are the most cultivated evergreen trees in the Mediterranean Basin, where they have deep historical and socioeconomic roots. The fungus *Verticillium dahliae* develops inside the vascular bundles of the host, and there are no effective applicable treatments, making it difficult to control the disease. In this sense, the use of integrated disease management, specifically the use of resistant cultivars, is the most effective means to alleviate the serious damage that these diseases are causing and reduce the expansion of this pathogen. In 2008, the University of Cordoba started a project under the UCO Olive Breeding Program whose main objective has been to develop new olive cultivars with high resistance to Verticillium wilt. Since 2008, more than 18,000 genotypes from 154 progenies have been evaluated. Only 19.9% have shown some resistance to the disease in controlled conditions and only 28 have been preselected due to their resistance in field condition and remarkable agronomic characteristics. The results of this study represent an important advancement in the generation of resistant olive genotypes that will become commercial cultivars currently demanded by the olive growing sector. Our breeding program has proven successful, allowing the selection of several new genotypes with high resistance to the disease and agronomical performance. It also highlights the need for long-term field evaluations for the evaluation of resistance and characterization of olive genotypes.

## 1 Introduction

Olive trees are a major crop in the Mediterranean Basin and in other new olive-growing countries, such as Australia, Argentina and Chile. Spain, with 2.62 million ha, is the main producer, contributing approximately 45% and 23% of total oil and table olive production, respectively (FAOSTAT, 2021). Verticillium wilt, caused by the soil pathogen *Verticillium dahliae* Kleb., is the most threatening fungus disease for this crop worldwide. Over the last two decades, *V. dahliae* has killed thousands of olive trees worldwide, with mortality exacerbated by the presence of susceptible cultivars, the use of irrigation and plantation on soils previously cultivating annual crop hosts of the pathogen, such as cotton or tomato (Montes-Osuna and Mercado-Blanco, 2020).

The lack of effective chemical control of the disease, the susceptibility of the most important olive cultivars, and the long persistence of the fungus in infested soil have motivated the search for a resistant germplasm as part of a fundamental strategy for the integrated control of the disease (Lopez-Escudero and Blanco-Lopez, 2007).

For this purpose, the resistance of a wide range of olive cultivars has been tested in recent decades (García-Ruiz et al., 2014; García-Ruiz et al., 2015; Godena et al., 2022; Markakis et al., 2022). This screening is still ongoing, and to date, hundreds of cultivars have been evaluated worldwide. Unfortunately, most cultivars have proven to be susceptible to *V. dahliae* infections (Lopez-Escudero and Mercado-Blanco, 2011). Only three cultivars, ‘Empeltre’, ‘Frantoio’ and ‘Changlot Real’, have shown a high level of resistance to Verticillium wilt. However, these cultivars present significant agronomical disadvantages, which have discouraged their use in affected orchards (Lopez-Escudero et al., 2004; Lopez-Escudero and Blanco-Lopez, 2007; Martos-Moreno et al., 2006; Trapero et al., 2013a; García-Ruiz et al., 2014; García-Ruiz et al., 2015; Trapero et al., 2015). For instance, ‘Empeltre’ has serious rooting problems; ‘Changlot Real’ is androsterile and susceptible to frost; and ‘Frantoio,’ the most widespread cultivar due to its resistance to the disease, presents a long unproductive period, excessive vigor and frost susceptibility (Rallo et al., 2005). For these reasons, the breeding of new olive cultivars resistant to Verticillium wilt has become necessary.

In 2008, under the framework of the University of Cordoba (UCO) olive breeding program, a breeding pipeline was developed and primarily focused on the selection of new olive cultivars highly productive and resistant to *V. dahliae*. This new breeding line benefited from the optimized methodology of crossbreeding cultivars, seed germination, forced growth, the reduction of the plant juvenile period and selection strategies already developed by the UCO breeding program since 1990 (Rallo et al., 2018). Specifically, the plant inoculation method with *V. dahliae* was optimized, with the root-dipping inoculation of five-week-old olive seedlings being the most effective inoculation method, allowing for the evaluation of thousands of seedlings (Trapero et al., 2013a).

In the first breeding approach, progenies obtained by the open pollination (OP) of olive cultivars, wild olive genotypes and other *Olea* species and subspecies (*Olea europaea* subsp. *cuspidata* and *Olea exasperata*) were screened with three objectives: first, to select Verticillium wilt-resistant genotypes; second, to characterize the relationship between the genitors and the distribution of resistant and susceptible genotypes among their offspring; and third, to identify the most suitable genitors to improve breeding for resistance to *V. dahliae*. In this work, a limited range of compatible crosses among the three resistant cultivars was detected (Trapero et al., 2015), with most of the crosses being repetitively unsuccessful, presumably due to incompatibility phenomena (Breton et al., 2016; Saumitou-Laprade et al., 2017). ‘Frantoio’ emerged as the best genitor to breed olive genotypes with increased resistance to Verticillium wilt as well as some wild olives and *O. exasperata* species. High genetic variability in the progeny response and resistance patterns compatible with quantitative inheritance and transgressive segregation were observed, even from nonresistant genitors (Arias-Calderon et al., 2015; Trapero et al., 2015; Valverde et al., 2021b).

We present the long-term results of this evaluation protocol and its efficiency for selecting olive genotypes resistant to Verticillium wilt, first under controlled conditions and later in highly infested soils over several years, with an additional field evaluation with replicates of the best genotypes. This study presents the first long-term results of a breeding program specifically designed for selecting new olive cultivars resistant to Verticillium wilt.

## 2 Material and methods

In this study, we performed different crosses between cultivars (Phase 0, P0), and then we evaluated the resistance of the progenies to Verticillium wilt in controlled conditions (Phase 1, P1). The selected resistant genotypes were then evaluated under infested field conditions (Phase 2, P2), where the most resistant and agronomically outstanding genotypes were selected, propagated and evaluated at the multilocal scale (Phase 3, P3).

### 2.1 Phase 0. Parental selection and crosses

The cultivars ‘Frantoio’, ‘Changlot Real’ and ‘Empeltre’, considered resistant to Verticillium wilt, were selected as principal genitors (Trapero et al., 2013b; García-Ruiz et al., 2015). Cultivars ‘Picual’, ‘Arbequina’, ‘Arbosana’ and ‘Koroneiki’ were also used as genitors combined with resistant cultivars to provide valuable agronomic characteristics, such as high oil content, fruit load, oil quality or early production (Diez et al., 2016; Barranco, 2017).

Controlled and open pollination crosses were conducted annually over almost a decade from 2008 to 2017. Directed crosses were performed in the spring by applying male pollen to previously bagged branches with flowers according to Rallo et al. (2018). In total, 154 crosses were performed, and 13,892 new genotypes were evaluated for resistance to *V. dahliae* (**Table 1**).

**Table 1:** Number of genotypes generated (Phase 0) and evaluated in controlled conditions (Phase 1) in the olive breeding program and corresponding phytopathological parameters.

Year of	Progenies	Sown seeds	Germinated seeds	Inoculated genotypes	Selected genotypes		Final	Incidence	Mortality	RAUDPC
crosses/inoculation	(n°)	(n°)	(n°)	(n°)	(n°)	(%)	Severity	(%)	(%)	(%)
2008/09	8	412	282	137	33	24.1cd	2.0cde	75.2bc	32.1c	30.6
2009/10	45	3,853	2,450	1,829	301	16.5cde	1.8de	56.9cd	33.7c	21.4
2010/11	26	4,940	3,096	3,324	419	12.6cde	1.5de	63.8bcd	13.2de	20.5
	23	3,162	2,616	2,365	184	7.8e	1.3de	63.5bcd	10.6de	-^2^
2011/12	36	3,676	2,924	1,953	195	10.0de	2.5bc	81.0b	41.0bc	32.8
	2	104	72	32	0	0.0f	4.0a	100.0a	96.7a	81.3
2013/14	12	2,900	1,705	916	224	24.5cd	1.9cde	72.9bc	31.0c	-^2^
	89	2,596	1,902	1,155	195	16.9cd	2.7bc	76.5bc	57.6b	33.7
2014/2015	9	728	421	240	40	16.7cd	2.5bc	80.4b	35.0bc	27.4
	6	884	636	466	151	32.4bc	2.1bce	63.4bcd	36.4bc	-^2^
2015/2016	24	2,392	1,400	990	761	76.9a	0.5f	22.6e	5.6e	7.5
2016/2017	18	1,768	880	485	255	52.6b	1.3e	46.8de	23.5cd	23.8
Average	154^1^	27,415	18,384	13,892	2,758	19.9	1.8	63.8	25.6	23.9

^1^Number of different progenies.

^2^Seedlings evaluated only for the final severity.

Values followed by the same letter are not significantly differentaccording to Least Significant Difference (LSD) test at P = 0.05.

### 2.2 Phase 1. Germination, seedling screening and forced growth

**Figure 1:**
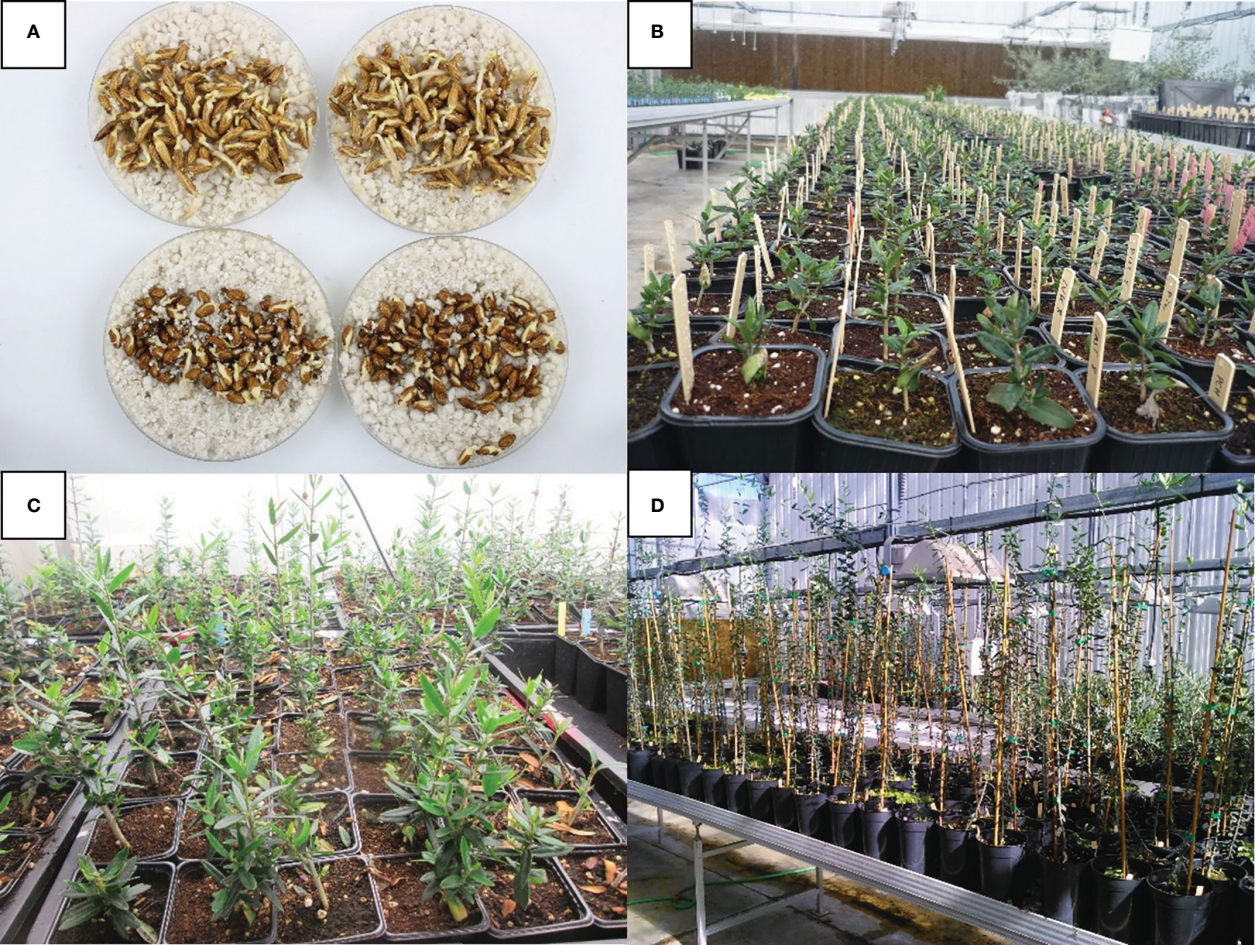
Phase 1. University of Cordoba Verticillium Olive breeding program **(A)** Seed germination; **(B, C)** seedling in *V. dahliae* evaluation in greenhouse; **(D)** resistant genotypes ready to be planted in the experimental fields.

*Germination.* The fruits, derived from the directed crosses, were harvested each year between September 15 and October 31. Naked seeds were stratified in pot trays filled with a mix of blond peat moss, coconut fiber, substratum and perlite at 13 to 14°C, R.H. = 95% in the dark in a climatic chamber (**Figure 1A**).

*Inoculation.* Olive seedlings were inoculated 40 days after germination, when they were 7 cm high and had two to three pairs of real leaves, by dipping their bare root systems for 30 min in a suspension of 10^7^ conidia/ml of the isolate V117 of *V. dahliae*, a highly virulent cotton defoliating isolate (Lopez-Escudero et al., 2004; Trapero et al., 2013a). Inoculated seedlings were transplanted in pots and arranged in greenhouse benches according to a completely randomized block design with a different number of plants per progeny (**Table 1**, **Figures 1B, C**). Seedlings were incubated for 15 weeks, with 16 h of light/day and temperatures of 23 ± 2°C (day) and 18 ± 2°C (night). Control seedlings were treated following the same procedure but dipping their roots in sterilized distilled water.

*Disease progress and selection of resistant genotypes.* To assess the progress of Verticillium wilt in the seedlings, disease severity was evaluated using a 0 to 16 rating scale. The scale estimated the percentage of tissue affected by wilted leaves, chlorosis, defoliation and/or necrosis using four main categories or quarters (<25, 26-50, 51-75, and 76-100%) with four values per category (Valverde et al., 2021a). Thus, each scale value represents the number of sixteenths of affected plant area. The scale values (X) were linearly related to the percentage of affected tissue (Y) with the equation Y = 6.25X - 3.125. The relative area under the disease progress curve (RAUDPC) was obtained from the severity values by applying the following formula based on Campbell and Madden (1990):


$$RAUDPC=\frac{100}{\left(s_{max}\times t_{e}\right)}\times \sum_{i=1}^{n}\frac{\left(s_{i}+s_{i+1}\right)}{2}\times (t_{i+1}-t_{i})$$

where *s_i_* = the disease severity value for the evaluation number *i*; *s_max_* = the maximum value of severity; *t_i_* = the number of days from planting to evaluation *i*; *t_e_* = the total length of the evaluation period in days; and *n* = the number of evaluations.

We also calculated the percentage of affected plants or disease incidence and the percentage of dead plants or mortality for each progeny. All of these parameters were used, together with RAUDPC values, as additional data to assign the resistance level of the genotype according to Lopez-Escudero et al. (2004) and Trapero et al. (2013a). Plant vegetative growth was measured every two weeks. Subsequently, only genotypes showing no symptoms during the evaluation period and able to consistently grow more than 3 cm after inoculation were selected (Trapero et al., 2013a).

Nearly five percent of the inoculated seedlings were randomly sampled to assess their infection by the pathogen. Seedling stems were washed in running tap water and surface disinfected in 0.5% sodium hypochlorite for 45 seconds. Stem pieces were placed on potato dextrose agar (PDA) plates and incubated at 24°C in the dark for 6 days.

*Forced growth.* The growth of the seedlings selected as resistant was forced following the protocol optimized by Santos-Antunes et al. (2005). In summary, plants were grown in a greenhouse with permanent lighting using high-pressure sodium vapor lamps, with an average temperature of 26°C (min 15°C and max 30°C), and under fertigation. During this period, the lateral shoots of the plants were clipped until the plants reached 80-100 cm high when they were planted in the field (**Figure 1D**).

### 2.3 Phase 2. *Verticillium dahliae*-infested field evaluation of selected seedlings

*Plant material.* The seedling genotypes evaluated in P2 were obtained and selected as described in Section P1. The number of genotypes per cross was variable (**Table 1**). To estimate the efficiency of the seedling inoculation methods under field conditions, in the Trajano field trial (P2-2), we compared the resistance of genotypes to four progenies (‘Changlot Real’ OP, ‘Frantoio’ OP, ‘Picual’ x ‘Arbequina’ and ‘Picual’ x ‘Frantoio’), which were selected as described in P1 like resistant, together with genotypes that were used as noninoculated control cultivars (genotypes that had not been previously inoculated in P1). The selection method (inoculated/control noninoculated) was considered a treatment for the genotypes in which they were applied, and therefore, they were equally distributed among the described blocks, with 20 genotypes included per selection method (**Table 2**).

**Table 2:** Location of the field trials of Phase 2 (P2) and their planting year, inoculum density, number of evaluated genotypes, incidence and mortality.

Field	Location	Year of plantation	Inoculum density^a^	Genotypes (n°)	Selected genotypes		Incidence (%)	Mortality (%)
					(n°)	(%)		
P2-1	Guadalcazar (Cordoba)	2011	1.5	317	3	0.95	13.1	9.6
		2012						
P2-2	Trajano (Sevilla)	2012	21	296	6	2.03	40.9	30.7
		2013						
P2-3	Villanueva de la Reina (Jaén)	2014	37	656	19	2.97	29.4	23.3

a Microsclerotia/gr of soil. Inoculum density was assessed by wet sieving with Modified Polypectate Sodium Agar media with 20 replications (plates) per analysis.

*Field plantation and experimental design*. After forced growth, resistant seedlings generated between 2008 and 2013 were progressively planted in three experimental fields naturally infested with *V. dahliae* (**Table 2**). These fields were chosen based on their inoculum density in the soil and their previous history of susceptible crops to the disease. All plantations in P2 were in the Guadalquivir Valley (andalucía, southern Spain). A total of 317, 296 and 656 genotypes were planted in each trial, respectively (**Table 2**).

For all orchards, a) the distance between rows and trees within rows (m) was set to 4 × 1.5 m; b) plants were drip irrigated with an annual dosage of 1,500 m^3^/ha per year; and c) the cultivars ‘Picual’ (susceptible), ‘Arbequina’ (moderately susceptible) and ‘Frantoio’ (resistant) (Lopez-Escudero et al., 2004; Trapero et al., 2013b) were planted as control cultivars of the disease for its known resistance to disease, tree vigor and agronomical performance. Control cultivars and progenies were arranged in a randomized block design using genotypes from each progeny as a homogeneous group, with 4 blocks and a variable number of plants per block: for control cultivars, at least 3 replicates were planted per block, while for the selected genotypes, the number of plants was different depending on the number of resistant genotypes in each progeny distributed among the 4 blocks.

*Disease assessment.* Evaluation of the disease was performed following the procedure described in P0 during artificial inoculations but adapted to the size of plants in field conditions. Fields were monitored every five weeks to evaluate disease symptoms. The evaluations were more frequent during the most favorable periods for disease development: the spring, early summer and fall. All of the affected plants were sampled to confirm the presence of the pathogen in symptomatic tissues as described above.

*Agronomic characteristics and genotype selection.* Once plants reached the adult stage, flower and crop load values were evaluated in the spring and autumn, respectively. Evaluations were conducted over at least 2 years of production using a visual scale of 0 (no load) to 3 (high load). Oil content was also determined using an NMR fat analyzer and expressed as a percentage on both fresh and dried weight bases using NMR analyzer Minispec NMS100 (Bruker Optik GmbH, Ettlingen, Germany) (Vlahov, 1999). Finally, the architecture of the trees was evaluated according to the growth habit, canopy density, trunk diameter and height of the plant. Height was measured with a meter, and trunk diameter was measured using an electronic gauge. We evaluated the growth habit visually as upright, spreading or drooping. For the evaluation of canopy density, we used a scale where 1=dense, 2=moderate and 3=sparse. The most promising genotypes were selected based on the complete absence of Verticillium wilt symptoms, early flowering, high production and oil content. These features were always compared to those shown by the control cultivars (‘Picual’, ‘Arbequina’ and ‘Frantoio’).

### 2.4 Phase 3. Propagation and agronomic field evaluation of the selected genotypes

We selected the best genotypes according to their performance under field conditions. These genotypes were clonally propagated by soft cuttings in a propagation chamber (Barranco, 2017). The propagated plants were grown in a greenhouse for at least six months by applying forced growth techniques described elsewhere until they were approximately 80 centimeters high and were planted in the field.

**Figure 2:**
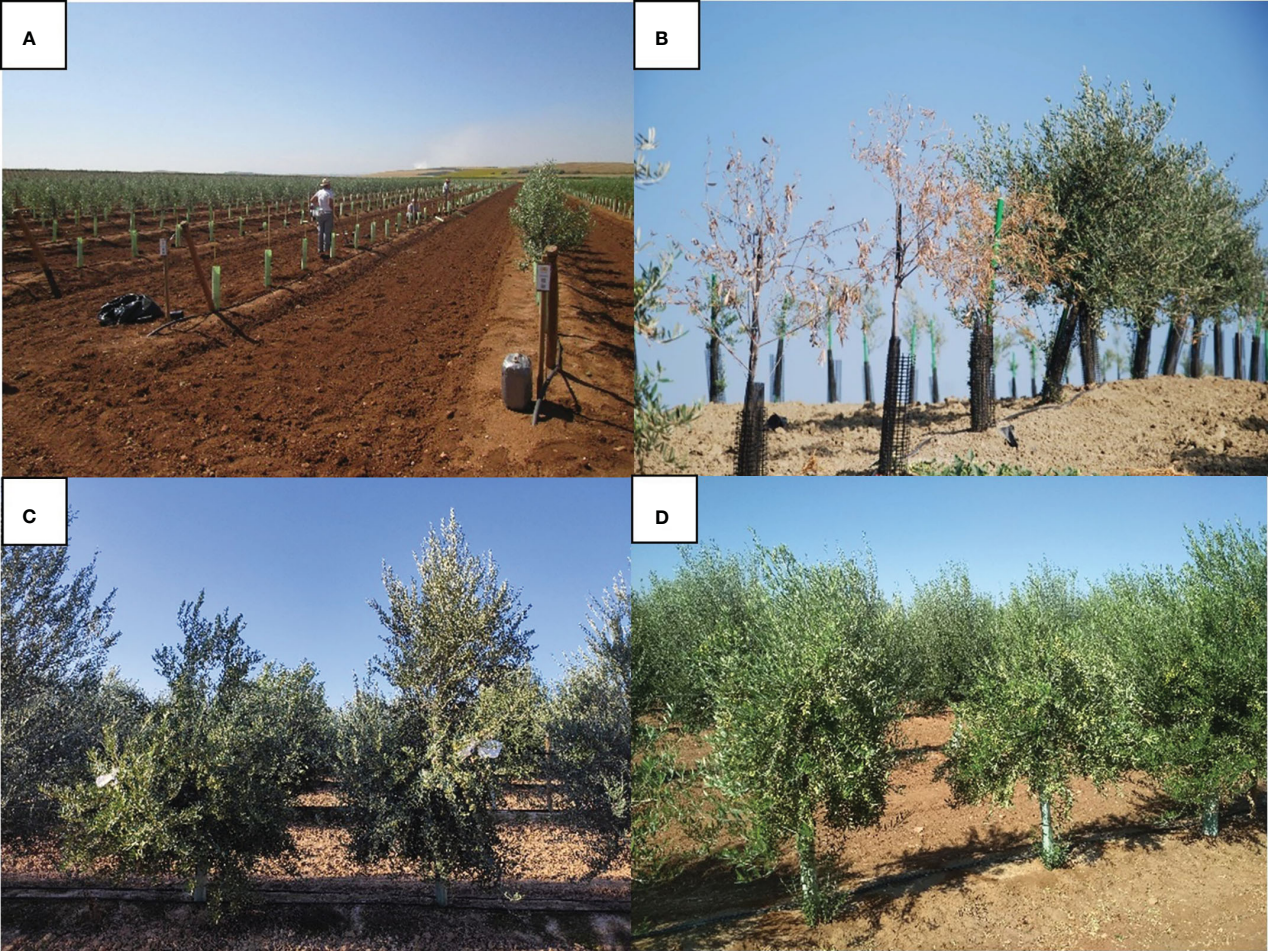
Phase 3: **(A)** plantation day; **(B)** ‘Picual’ (left) and ‘Frantoio’ (right) cultivars in evaluation in the experimental field; **(C, D)** genotypes selected and planted with repetitions.

For P3, we set up three field experiments in different locations. Two of them, occurring in Arjona (P3-1) and Villanueva de la Reina (Jaén province) (P3-2), with naturally infested soils with *V. dahliae*, involved 5 and 37 propagules per gram of soil, respectively. The third experimental field in Carmona, Sevilla (P3-3) included soil free of the pathogen. The aim of this latter experiment was to evaluate genotype performance under no biotic stress and optimal growing conditions. In the three experiments, plants were arranged in four blocks with 4 replicates of each genotype per block in addition to the cultivars ‘Picual’, ‘Frantoio’, ‘Arbequina’ and ‘Arbosana’ used as control cultivars (**Figure 2**).

The symptoms caused by *V. dahliae* were evaluated every 5 weeks. We also evaluated the following agronomic traits: the vigor (including height, width and trunk diameter in the winter), flowering and fruit load and oil content of the fruits. The evaluation methods for these characteristics are described in Section 2.2. In addition, olive oil from the evaluated genotypes was extracted and characterized. To do so, two kg of fruit from each block was manually harvested by sampling all orientations within the canopy of 4 trees per block. In total, 4 samples were harvested per genotype (one sample per block). Sampling was performed when the fruits were at a ripening index (RI) of 2.0 (yellowish-red color), according to the method proposed by the International Olive Oil Council (International Olive Council, 2011), from October to December. Monovarietal virgin olive oils were obtained using an Abencor extraction system (MC2 Ingeniería y Sistemas, Sevilla, Spain) under optimized conditions following the process described by Miho et al., 2018. Then, the samples were stored in amber glass bottles at –18°C until analysis.

Oil fatty acid profiles were characterized by gas chromatography (Waktola et al., 2020). We also measured the stability to oxidation of the oil samples by applying the Rancimat method (Tinello et al., 2018).

### 2.5 Statistical analysis

The agronomical data, including fresh and dry oil content, oleic acid content, stability to oxidation, height, width, trunk diameter, growth habit and canopy density, were analyzed by one-way analysis of variance (ANOVA). An ANOVA of the RAUDPC data using a randomized complete block design was performed to analyze differences between all of the progenies and cultivars in their resistance to Verticillium wilt that were compared between them by Fisher’s protected LSD test at *P* = 0.05. The variances fulfilled the requirements for homogeneity according to the Levene, Obriene, and Brown–Forsythe tests. A two-factor full factorial ANOVA design was also performed to analyze the differences between selection methods, with the two factors being inoculation/noninoculation and progeny. Original data were suitable for ANOVA without transformation, and mean values of the progenies and cultivars were compared by the LSD test at P = 0.05. For all of the experiments, incidence and mortality values were analyzed by means of Pearson’s chi-square test at P = 0.05. All analyses were performed using the Statistix 10.0 program (Analytical Software, Tallahassee, FL, USA).

## 3 Results

### 3.1 Phases 0 and 1. Seedling germination and genotype disease evaluation

From 2008 to 2017, a total of 27,415 olive seeds from 154 progenies, derived from cultivars in free pollination and directed crosses, were sown with an average germination rate of 67.05%. In total, 13,892 genotypes were inoculated, and 2758 of them were selected for their resistance (19.9%). Regarding the phytopathological parameters, the mean of the final severity of symptoms in the inoculated plants was 1.8, with an average incidence, mortality and RAUDPC of 63.8, 25.5 and 23.9%, respectively (**Table 1**).

### 3.2 Phase 2. Field evaluation: One plant per genotype

The first symptoms of the disease were observed in all of the fields approximately six months after planting. The development of the disease was more rapid and extensive during the spring and autumn months, with occasional symptoms observed during the winter. The infected trees were usually killed a few weeks after the appearance of first symptoms. This pattern was particularly remarkable for the cultivar ‘Picual,’ which was used as a susceptible control. The fungus was consistently isolated from most of the symptomatic shoots that were sampled.

After four years of field evaluations, the average incidence values (percentage of affected genotypes) in experimental plots P2-1, P2-2 and P2-3 were 13.1%, 40.9% and 29.4%, respectively, with mortality values ​​of 9.6%, 30.7% and 23.3%, respectively (**Table 2**). Regarding the control cultivars, ‘Picual’ reached mortality and incidence values ​​of higher than 70% in the P2-2 and P2-3 fields; in contrast, ‘Frantoio’ showed an average incidence of 22.6% and mortality of 1.3% with an incidence and mortality of 0 found in P2-1 (**Table 3**).

**Table 3:** Phytopathological parameters evaluated in the control cultivars in the three infested plots.

Cultivar	Incidence (%)				Mortality (%)			
	P2-1	P2-2	P2-3	Average	P2-1	P2-2	P2-3	Average
‘Picual’	34.3a	90.3a	70.4a	89.1a	23.1a	87.1a	80.0a	63.4a
‘Arbequina’	29.4a	57.1b	41.3b	48.7b	23.5a	9.5b	12.5b	15.1b
‘Frantoio’	0b	38.5c	29.5c	22.6b	0b	4.0b	0.0c	1.3c

Values followed by the same letter are not significantly differentaccording to Least Significant Difference (LSD) test at P = 0.05.

**Figure 3:**
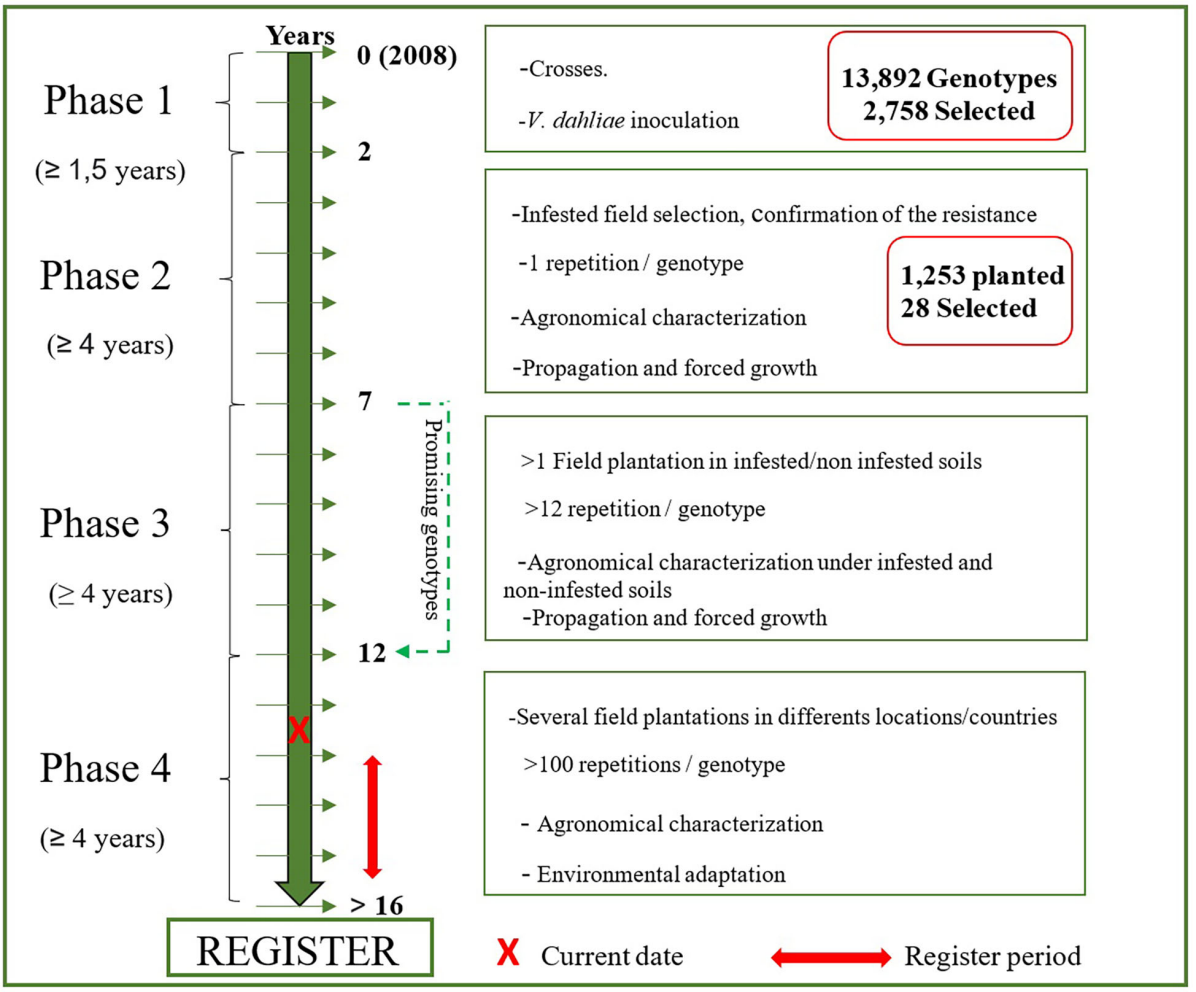
Steps and timeframe of the development of new cultivars resistant to *Verticillium dahliae* under the UCO Olive Breeding Program.

In summary, 86.9%, 59.1% and 70.6% of the new genotypes in plots P2-1, P2-2 and P2-3, respectively, did not show disease symptoms under field conditions. However, only 28 of 1,269 genotypes planted in the field combined this characteristic with valuable agronomic traits. These 28 genotypes represent 2.2% of the genotypes evaluated under field conditions and only 0.2% of the germinated seedlings (**Figure 3**).

The number of genotypes selected for their positive agronomic characteristics depended on the performance of the cultivars used as genitors. For instance, in experimental field P2-1, there was a lower percentage of selected genotypes than for the other two naturally infested plots. This difference was due to the use of some wild olives as parents, which exhibited late bearing and low oil content.

The selected genotypes always presented a similar or earlier bearing time and similar or higher oil content than the control cultivars. In field P2-2, it was not possible to compare with ‘Picual’ because the plants of this cultivar died before the first harvest due to the disease. In this case, we show agronomic data for the resistant cultivar ‘Changlot Real’ as a reference (**Table 4**).

**Table 4:** Agronomic characteristics of the selected genotypes and control cultivars after their evaluation in field conditions (Phase 2).

Field	Genotypes	Genitors (Cultivars)		Years to	Olive Oil	Olive Oil
		Female	Male	first	content	content
				flowering	on fresh^1^ (%)	on dry^2^ (%)
P2-1	1	Koroneiki	unknown	4	25.5	50.1
	2	Changlot Real	Empeltre	4	21.8	46.1
	3	Frantoio	Empeltre	4	20.3	46.3
	Picual	Control		4	14.9	38.4
	Arbequina			4	18.5	44.5
	Frantoio			4	20.6	44.5
P2-2	4	Changlot Real	Koroneiki	2	21.1	45.0
	5	Picual	Frantoio	3	30.3	55.3
	6	Picual	Frantoio	3	20.2	48.2
	7	Frantoio	Changlot Real	3	20.5	40.8
	8	Picual	unknown	2	19.9	44.0
	9	Koroneiki	Arbosana	2	19.3	39.2
	Changlot Real	Control		3	18.6	44.5
	Arbequina			3	17.5	38.2
	Frantoio			3	20.6	38.8
P2-3	10	Arbosana	Sikitita	1	18.9	48.9
	11	Arbosana	unknown	2	26.1	56.2
	12	Sikitita	Arbosana	2	19.1	43.1
	13	Arbosana	Sikitita	1	17.3	43.4
	14	Arbosana	Sikitita	2	20.4	53.6
	15	Sikitita	Arbosana	3	18.1	45.7
	16	Arbosana x Koroneiki	unknown	2	19.9	44.9
	17	Arbosana x Koroneiki	unknown	3	21.3	45.4
	18	Arbosana	Sikitita	3	18.0	42.5
	19	Arbosana	Sikitita	1	16.4	43.2
	20	Sikitita	Arbosana	2	19.9	43.9
	21	Arbosana	Sikitita	3	20.3	44.3
	22	Arbosana	Sikitita	4	18.5	43.9
	23	Arbosana x Koroneiki	unknown	4	19.5	45.2
	24	Arbosana	unknown	4	21.6	42.2
	25	Gemlik	unknown	2	23.6	53.9
	26	Frantoio	Arbequina	4	23.8	50,1
	27	Itrana	unknown	3	19.1	46.9
	28	Frantoio	unknown	3	20.5	49.3
	Picual	Control		3	17.0	42.0
	Arbequina			1	18.8	42.9
	Frantoio			3	19.9	42.5

^1^Olive oil content matter on fresh. Mean of two seasons values.

^2^Olive oil content matter on dry. Mean of two seasons values.

### 3.3 Efficiency of the inoculation method in selecting for field resistance

The three disease parameters evaluated were significantly lower for the genotypes selected by root dip inoculation. The factorial analysis revealed no significant interaction between the selection method (inoculated/control) and the progeny (P = 0.95); therefore, the selection method was effective for all of the progenies (**Table 5**). On the other hand, differences between progenies were not significant (P = 0.11).

**Table 5:** Disease parameters of the genotypes evaluated in field trial P2-2. Genotypes were previously either selected for resistance to *V. dahliae* or not selected (used as controls) in the seedling stage.

Inoculation treatment^a^	RAUDPC^b^	Incidence (%)^c^	Mortality (%)^c^
Conidial suspension by root-dip	18.7 a	35*	20*
Control (Water)	45 b	65	50

a Olive crosses used were: ‘Changlot Real’ OP, ‘Frantoio’ OP, ‘Picual’ x ‘Arbequina’ and ‘Picual’ x ‘Frantoio’. The number of plants from each inoculation treatment was the same for each cross. Root dip inoculation had been performed using a conidial suspension of a highly virulent *V. dahliae* isolate.

b Values were estimated 2 years after planting and they are the mean of 20 genotypes per inoculation treatment. Values followed by the same letter are not significantly different according to Least Significant Difference (LSD) test at P = 0.05.

c Values followed by an asterisk (*) correspond to treatments whose % of incidence or mortality was significantly lower than the control genotypes (genotypes which had not been selected for resistance), according to Chi-Square test at P = 0.05.

### 3.4 Phase 3. Clonal propagation and field evaluations: Several replicates per genotype

In total, 28 genotypes selected in P2 were propagated by soft cuttings, and when they were approximately 80 cm tall, they were planted in three experimental fields (P3-1, P3-2 and P3-3). After growing for 4 years, these new resistant genotypes did not show symptoms of the disease in P3-1 and P3-2. In P3-3, genotypes n° 4-9 (**Table 4**) showed higher or similar oil levels than the control cultivars (**Table 6**). Regarding the percentage of oleic acid, ‘Picual’ showed the highest value of 78.6%, followed by the genotypes ‘n° 5’ (76.5%), ‘n° 9’ (75%) and ‘n° 7’ (73.6%). These cultivars also presented high levels of oil stability. ‘Picual’ was the most stable cultivar, with an average of 131.9 hours. On the other hand, the control cultivars ‘Frantoio,’ ‘Arbosana,’ and ‘Arbequina’ and the genotype ‘n° 6’ showed the lowest values ​​for this parameter at 30.4, 49.2, 55.6 and 55.1 hours, respectively. Regarding vigor, the genotypes with less vigor, according to their height and trunk diameter, included the variety ‘Arbosana’ and genotype ‘n° 9,’ which comes from the cross ‘Koroneiki’ x ‘Arbosana.’ On the other hand, control cultivars ‘Frantoio’ and ‘Picual’ and genotypes ‘n° 4,’ ‘n° 7’ and ‘n° 8’ showed the highest vigor values (**Table 6**).

**Table 6:** Agronomic characteristics evaluated in the selected genotypes and control cultivars in trials P3-3.

Genotype	Months to first flower	Oil on fresh (%)	Oil on dry (%)	Oleic acid (%)	Stability (hours)	Height (cm)	Width (cm)	Ø (mm)	Growth habit	Top density
‘Arbequina’	19.2d	15.6def	39.2de	64.3f	55.6cd	262.9c	182.9bcd	54.8b	1.8abc	1.5cd
‘Picual’	24.0c	15.9de	39.6de	78.6a	131.9a	285bc	188.8bc	58.1b	1.4cd	1.6bcd
‘Frantoio’	36.0b	20.5b	45.0b	64.8f	30.4e	288.13ab	192.5b	62.8ab	1.3d	1.5cd
‘Arbosana’	17.6d	13.6g	37.1e	70.3de	49.2d	225.9d	161.0f	44.9c	2.0a	1.4de
4 ‘Changlot Real’ x ‘Koroneiki’	24.0c	15.5ef	40.2cd	71.2d	75.6b	297.5ab	195.0ab	57.8b	1.6abcd	1.9abc
5 ‘Picual’ x ‘Frantoio’	43.6a	24.6a	53.9a	76.5b	76.4b	262.0c	166.0ef	40.95c	1.4bcd	2.1a
6 ‘Picual’ x ‘Frantoio’	19.4d	16.7cd	43.1b	69.2e	55.1cd	306.9ab	178.2cde	67.8a	1.9ab	1.8abc
7 ‘Picual’ OP	23.4c	14.3fg	31.3e	62.6g	65.9bc	312.3ab	171.8def	58.1b	1.7abcd	1.7abcd
8 ‘Frantoio’ OP	26.0c	17.5c	39.2bc	73.5c	71.8b	316.6a	208.4a	62.6ab	1.8abc	2.0ab
9 ‘Koroneiki’ x ‘Arbosana’	23.4c	16.7cde	39.3de	75.0bc	74.2b	218.6d	158.5f	45.4c	2.0a	1.0e

Values followed by the same letter are not significantly different according to LSD testing at P = 0.05

## 4 Discussion

### 4.1 Variability and selection in controlled conditions

Currently, *V. dahliae* causes the main fungal disease that affects olive trees in the Mediterranean region due to a lack of specific control measures for its management (Blanco-Lopez et al., 1984; Montes-Osuna and Mercado-Blanco, 2020). In integrated management, one of the main tools involves the use of resistant cultivars (Lopez-Escudero and Mercado-Blanco, 2011). However, due to the few existing resistant cultivars in olive germplasm (García-Ruiz et al., 2014; García-Ruiz et al., 2015), it is necessary to develop resistant cultivars that are also adapted to new olive growing conditions, which require low vigor, among other characteristics. Some olive cultivars and wild forms have been evaluated as genitors for obtaining new resistant olive cultivars (Colella et al., 2008; Trapero et al., 2015; Serrano et al., 2021). However, there is still vast genetic diversity to be explored within the species (Diez et al., 2015).

Over more than a decade, the breeding program carried out at the UCO has been working on obtaining new olive cultivars resistant to Verticillium wilt following the three-step protocol described in this manuscript, which combines directed crosses, evaluations in controlled and field conditions and the progressive selection of outstanding genotypes. Since 2008, 13,892 genotypes have been evaluated for *V. dahliae* under this program. The number of resistant genotypes per progeny basically depended on the level of resistance of the parents (Trapero et al., 2015; Valverde et al., 2021b). For this reason, the percentage of resistant genotypes in progenies ranged between 0 and 76%. However, as previously reported, it was possible to find resistant genotypes even in progenies with high average susceptibility, such as ‘Arbosana’ x ‘Sikitita’ (Trapero et al., 2015; Valverde et al., 2021b). The opportunity to select resistant genotypes from susceptible cultivars or progenies broadens the genetic pool available for breeding olives for Verticillium wilt resistance, but widespread screening of seedlings will be necessary to find resistant genotypes among these progenies.

Many genes are involved in the resistance of olive plants to *V. dahliae* (Jiménez-Ruiz et al., 2017; Leyva-Pérez et al., 2018; Serrano et al., 2020). This type of polygenic resistance has also been corroborated for this disease in crops such as cotton (Bolek et al., 2005; Xiong et al., 2020) and strawberry (Zebrowska et al., 2006), which makes obtaining genotypes highly resistant to the disease much more complicated. Besides that, recent works show the importance of a broad vision of resistance, not only at the specific level of general genetic resistance, but also of the importance of a holistic/multilevel perspective, which can indirectly determine the resistance of the variety. In the same way, this resistance based on the functional traits of the root can also determine the level of general resistance of each variety (Cardoni et al., 2022). In this sense, the resistance to *V. dahliae* in olive may be determined by the soil microbiota, although currently knowledge of these relationships is quite low (Fernandez-Gonzales et al., 2020).

In this work there was variability in the average resistance levels of the same progeny between years of evaluation in controlled conditions, probably due to inherent experimental variability and its influence on the development of the pathogen. This observation is also in agreement with previous studies conducted in controlled conditions. For instance, Trapero et al. (2015) found high variability in mortality values ​​obtained when evaluating genotypes from ‘Arbequina’ x ‘Picual,’ with values ​​ranging from 9.3% to 71.7% in controlled conditions. García-Ruiz et al. (2014, 2015) also reported different mortality values even for the same cultivar, ‘Picual,’ ranging from 33.3% to 71.4% under controlled conditions. This variability in results reinforces the need to corroborate the potential resistance of the genotypes in long-term field conditions, that is, in naturally infested soils with different levels of inoculum (Trapero et al., 2013a). In addition, the agronomical performance of the new cultivars should also be evaluated in different environments to test their stability and consistency (Navas-Lopez et al., 2019).

### 4.2 Influence of the inoculation method on the selection of resistant genotypes

Genotypes that had been selected for Verticillium wilt resistance before planting in the infested field by root dip inoculation were more resistant than genotypes that had been used as control cultivars and had not been selected. As the interaction between progeny and selection methods was not significant, we can confirm that the inoculation method was effective in screening for resistant genotypes regardless of the genitors used. On the other hand, resistance mechanisms working before the entrance of the conidia into the root xylem are also overlooked when using the root dip inoculation method; therefore, an efficient method involving the infection of plants by microsclerotia would be of much interest, as has been developed for other species (Steventon et al., 2002; Bae et al., 2011). However, these methods usually require more time than root dip inoculation to observe symptoms.

Plants inoculated with *V. dahliae* in the seedling stage and then selected for their resistance after several weeks without symptoms may express induced resistance during an unknown period of time. Induction of resistance has been reported for a wide range of crops and pathogens (Durrant and Dong, 2004), including Verticillium wilt of olive (Lopez-Moral et al., 2022). Martos-Moreno (2003) reported that infection with a nondefoliating isolate can induce more resistance to subsequent inoculation with a defoliating isolate under controlled conditions. Considering this, in this study, we might be overestimating the level of resistance in genotypes inoculated with *V. dahliae* in the seedling stage. However, after seedling inoculation, plants were subjected to forced growth (Santos-Antunes et al., 2005) for 7 months, and afterward, they were evaluated for more than 24 months in *V. dahliae*-infested field conditions. Thus, the presence of induced resistance so long after inoculation is not very likely.

The opportunity to select resistant genotypes from susceptible cultivars or progenies broadens the genetic pool available for breeding olives for Verticillium wilt resistance, but massive screening of seedlings would be necessary to find resistant genotypes among these progenies.

### 4.3 Selection of resistant genotypes under field conditions

The levels of resistance of the three control cultivars, ‘Frantoio,’ ‘Picual’ and ‘Arbequina,’ ranked in the same order as in previous field studies, with ‘Frantoio’ being the most resistant cultivar, ‘Picual’ being the most susceptible and ‘Arbequina’ showing intermediate behavior (Lopez-Escudero et al., 2004; Trapero et al., 2013b). All of the selected progenies were significantly more resistant than the control cultivar ‘Picual’ under field conditions in all of the experimental fields. This indicates the successful selection of cultivars used as genitors but mainly the effectiveness of the screening methods for selecting resistant genotypes, given that it was possible to find resistant genotypes derived from susceptible genitors.

The evaluation of the preselected genotypes in field conditions, i.e., heavily infested with *V. dahliae*, is a necessary and efficient means to breed cultivars for Verticillium wilt resistance. In fact, only 638 genotypes (67%) of 952 initially planted in fields P2-2 and P2-3 remained free of symptoms after four years of evaluation. This fact highlights the importance of confirming resistance at the field level after evaluations under controlled conditions, since 33% of the genotypes selected as resistant under controlled conditions showed symptoms in the field evaluations, which may be due to the successive reinfections to which a plant is subjected in the field and to the multiple isolates present in soil (Lopez-Escudero and Mercado-Blanco, 2011).

Regarding agronomic characteristics, the control varieties confirmed their vigor values, with ‘Frantoio’ and ‘Picual’ being the most vigorous and ‘Arbosana’ being the most compact. These results are in line with those previously obtained (Tombesi et al., 2011; Diez et al., 2016), allowing us to make a fair comparison to new varieties under evaluation in the breeding program. Likewise, the highest oil fruit levels among the control varieties were obtained by ‘Frantoio’ and the lowest were shown by ‘Arbosana’, corroborating values obtained in other works (Barranco, 2017). When we observed the values of oleic acid content and oil stability, the control variety ‘Picual’ obtained the highest values ​​ (Barranco et al., 2017). The selected genotypes had agronomic values similar to those of commercial control varieties and therefore proved to be candidates for future resistant cultivars. However, it is necessary to continue evaluating the agronomic behavior of these genotypes under different environments and with a large number of control varieties. Even though the period for evaluating resistance to *V. dahliae* could be shorter than 4 years in highly infested soils, agronomic evaluation in a second phase with replicated genotypes requires a longer period, in line with other studies conducted to develop new olive cultivars (Leon et al., 2007; Zeinanloo et al., 2009; Ben Sadok et al., 2013). This second selection cycle in long-term field conditions is required to a) confirm the resistance of the selected seedlings and c) evaluate agronomical and phenotypical traits such as vigor, production or olive oil quality that will determine the agronomical value of these genotypes as new olive cultivars.

After 13 years from the first crosses and more than 27,000 seeds sown, only 28 genotypes have been selected due to their high level of resistance and agronomic behavior. This low rate is due to high selection pressure, mainly for disease resistance but also for good agronomic characteristics. Moreover, only a few of the genotypes will become new registered cultivars and might be part of the solution to the disease caused by *V. dahliae*, providing high crop and oil quality in areas affected by this disease. The results of this study represent an important advancement in the generation of resistant olive genotypes that will become the commercial cultivars currently demanded by the olive growing sector. Our breeding program has proven successful, allowing the selection of several new genotypes with high resistance to the disease and agronomical performance.

## Data availability statement

The original contributions presented in the study are included in the article/supplementary files. Further inquiries can be directed to the corresponding author.

## Author contributions

All authors conceived the experiment(s), PV and CT conducted the experiment(s), PV, CMD and CT analyzed the results. All authors reviewed the manuscript and approved it for publication.
